# Antibodies against H10N8 avian influenza virus among animal workers in Guangdong Province before November 30, 2013, when the first human H10N8 case was recognized

**DOI:** 10.1186/s12916-014-0205-3

**Published:** 2014-10-27

**Authors:** Wenbao Qi, Shuo Su, Chencheng Xiao, Pei Zhou, Huanan Li, Changwen Ke, Gregory C Gray, Guihong Zhang, Ming Liao

**Affiliations:** College of Veterinary Medicine, South China Agricultural University, Guangzhou, Guangdong Province 510642 People’s Republic of China; Division of Infectious Diseases and Duke Global Health Institute, Duke University, Durham, NC 27710 USA; Guangdong Center for Disease Control and Prevention, Guangzhou, People’s Republic of China

**Keywords:** Avian influenza virus, Seroepidemiological study, Poultry workers, Microneutralization

## Abstract

**Background:**

Considered an epicenter of pandemic influenza virus generation, southern China has recently seen an increasing number of human H7N9 infections. However, it is not the only threat. On 30 November 2013, a human H10N8 infection case was first described in China. The origin and genetic diversity of this novel virus is similar to that of H7N9 virus. As H10N8 avian influenza virus (AIV) was first identified from a duck in Guangdong Province during 2012 and there is also evidence of H10N8 infected dogs in this region, we sought to examine archived sera from animal workers to see if there was evidence of subclinical human infections before the first human H10N8 cases.

**Methods:**

We studied archived serum samples (cross-sectional study, convenience sample) collected between May and September 2013 from 710 animal workers and 107 non-animal exposed volunteers living in five cities of Guangdong Province. Study participants’ sera were tested by horse red blood cells (RBCs) hemagglutination inhibition (HI) and microneutralization (MN) assays according to World Health Organization guidelines. The A/Jiangxi-Donghu/346-1/2013(H10N8) virus was used. Sera which have an HI assay ≥1:20 were further tested with the MN assay. Questionnaire data were examined for risk factor associations with positive serological assays. Risk factor analyses failed to identify specific factors associated with probable H10N8 infections.

**Results:**

Among the 827 sera, only 21 animal workers had an HI titer ≥1:20 (18 had an HI titer of 1:20 and 3 had an HI titer of 1:40). None of these 21 subjects reported experiencing any influenza symptoms during the three months before enrollment. Among the three subjects with HI titers of 1:40, two had MN antibody titers of 1:40, and one had a MN antibody titer of 1:80 (probable H10N8 infections).

**Conclusions:**

Study data suggest that animal workers may have been infected with the H10N8 virus before the first recognized H10N8 human infection cases. It seems prudent to continue surveillance for H10N8 viruses among animal workers.

## Background

Located in southern China, Guangdong Province is home to some of the world’s largest populations of humans, chickens, ducks and pigs and has been associated with human outbreaks of severe acute respiratory syndrome (SARS) and highly pathogenic H5N1 avian influenza infections. This region of China has been considered an epicenter of novel influenza virus generation [1,2]. In recent years, a variety of novel swine and avian viruses have been detected in pigs and poultry in Guangdong Province [3-5]. The human H7N9 influenza strain, first detected in March 2013 [6], has quickly spread among poultry flocks in at least ten of China’s provinces, causing rapidly progressing lower respiratory tract infections in humans. As of 29 August 2014 at least 365 human infections have been identified (111 deaths) and have been reported to the World Health Organization [7]. In Southern China, an increasing number of human H7N9 infections have aroused public awareness of zoonotic avian influenza transmission but the novel H7N9 is not the only influenza problem. On 30 November 2013, the first human infection with H10N8 avian influenza virus (AIV) was found in a 73-year-old woman living in Nanchang City, China. She died nine days after the onset of illness. As of 15 February 2014, two additional human infections with H10N8 had been documented in Jiangxi Province, with one of them resulting in a second death [8]. The origins of the H10N8 viruses’ HA and NA gene segments were similar and thought to have moved first from wild birds to ducks and then to chickens. The six internal gene segments were similar to those of the H9N2 influenza viruses frequently detected in chickens [9]. Notably, the H10N8 virus emergence coincided with a second wave of the human H7N9 AIV outbreak [10], and subsequent to the human index case, more H10N8 AIV infections have been detected in both avian species and humans [3,11].

As H10N8 AIV was first identified in a duck from Guangdong Province in 2012 [3] and there is also evidence of H10N8 infected dogs in this region [12], it seems important to understand whether subclinical human infection with the H10N8 virus occurred before 30 November 2013. Hence, we conducted a retrospective cross-sectional, seroepidemiological study among animal workers in Guangdong Province.

## Methods

The animal worker sera were collected during the period June to August 2013 during a surveillance program for novel zoonotic influenza virus among animal workers living in five cities of Guangdong Province. Non-animal-exposed participants’ sera were similarly collected during the period May to August from middle school teachers and students in Guangzhou and Foshan cities who were healthy, reported no history of having received an influenza vaccine or having direct contact with swine or poultry, during the six months before enrollment. This study was approved by Guangdong Centers for Disease Control and Prevention and begun in early 2013. Study subjects were screened by telephone call and enrolled by informed consent. Participants completed a questionnaire which collected various relevant data including demographic, recent clinical signs and symptoms, and animal occupational exposure history.

Sera were first screened by a horse red blood cells (RBCs) hemagglutination inhibition (HI) assay against influenza virus A/Jiangxi-Donghu/346-1/2013 (H10N8). Sera with HI titers ≥1:20 were further studied with microneutralization (MN) assays against the same virus. It is important to note that horse RBCs show a high proportion of sialic acid α2,3-Gal binding, which is preferential for AIV. It had been observed that the use of horse RBCs significantly increased the sensitivity of detection of HI antibodies in the sera of confirmed H7N9 cases compared with the use of turkey RBCs, thus the World Health Organization recommends that horse RBCs should be used to detect HI antibodies for H7N9 virus infection [13]. So, we used the horse RBC HI assay to look for evidence of previous H10N8 virus infection.

Both assays were performed according to the World Health Organization guidelines [14]. During the HI assay, sera were pre-treated with receptor destroying enzyme and hemabsorbed with horse erythrocytes. Briefly, 25 μl of serial twofold dilutions of the treated serum samples were mixed with four hemagglutin in units (HAU) of virus in V-shaped microtiter plates and incubated at room temperature for 30 minutes. Then, 50 μl of 1.0% horse RBCs were added to each well and incubated at room temperature (22° to 25°C) for another 30 minutes. The HI titer was calculated as the reciprocal of the highest serum dilution that completely inhibited the hemagglutination of four HAU of virus. The MN assay was performed using two-fold dilutions in serum-freeDMEM starting at a dilution of 1:10. Then, 100 TCID_50_ of virus were added to the serially diluted serum at a 1:1 ratio (V/V) and incubated at 37°C for one hour. Finally, 0.2 ml of the virus-serum mixtures was transferred to 96-well monolayer plates and incubated in 5% CO_2_ at 37°C for 72 hours. Three wells were run for every dilution of each serum sample. We observed the pathogenic effects every day and tested the cell supernatants with a HA test to confirm the infection.

Study sera which had titers <1:20 in endpoint serum dilution against H10N8 antigens used in this study were considered to be negative. As there was no established HI titer standard to detect a mild or asymptomatic human H10N8 infection, and the positive-control samples have shown HI antibody titers ranging from 1:40 to 1:1280, in this seroepidemiological study we considered sera with HI titers above 1:40 as having possible evidence of previous H10N8 virus infection but only those sera with both HI and MN titers ≥1:40 as having probable evidence of previous infection with influenza A(H10N8) virus.

## Results

Archived sera and data from 827 poultry workers, poultry retailers in agricultural-trade markets, swine workers, veterinarians, slaughterhouse workers, zoo workers and non-animal-exposed volunteers were studied (Table 1). The total numbers of the 827 animal workers and non-animal-exposed volunteers participating in the current study were 223, 125, 175, 104 and 200 from Guangzhou, Foshan, Qingyuan, Jiangmen and Zhongshan cities, respectively. The age distribution in the five regions was comparable. About 30% of the participants were female and the majority of animal workers were swine workers (42.5%) or poultry retailers in agricultural-trade markets (23.5%). Interestingly, approximately 11% of participants reported having influenza symptoms during the last three months before enrollment.

**Table 1:** **Characteristics of study subjects upon enrollment, Guangdong China, 2013**

**Exposure variables**	**Total (number = 827)**	**Zoo workers (number = 48)**	**Swine workers (number = 352)**	**Slaughterhouse workers (number = 24)**	**Veterinarians (number = 26)**	**Poultry workers (number = 76)**	**Non-animal exposed controls (number = 107)**	**Poultry retailers in agricultural-trade (number = 194)**
Gender								
Female	253	11	109	0	0	18	45	70
Male	574	37	243	24	26	58	62	124
Age-group (years)								
< 20	30	2	4	0	0	1	19	4
20 to 30	160	19	53	2	4	14	18	50
30 to 40	228	18	93	6	8	26	17	60
>40	409	9	202	16	14	35	53	80
Mean age (years)	38.5	31.3	40.5	42.2	40.4	37.1	38.5	36.5

HI titers of ≥1:20 were detected in 21 (2.54%) of 827 study subjects (18 with a HI titer of 1:20 and 3 with a HI titer of 1:40). None of these 21 participants (all animal workers) reported having influenza symptoms during the three months before enrollment. Three of these 21 subjects also had MN antibody titers ≥1:40 against H10N8 AIV (Table 2) and were considered as having probable evidence of H10N8 infection: two with MN titers of 1:40 and one with a MN titer of 1:80. Occupations among the three (0.36%) of the 827 subjects with probable infections included two poultry workers and one veterinarian living in Guangzhou city, Guangdong Province (Table 1). Each had daily poultry exposure. No demographic, clinical or specific animal exposure risk factor associations were found in the three subjects with probable infections.

**Table 2:** **Characteristics of the study subjects whose sera were reactive against avian H10N8**

**Number**	**Age**	**Collect day**	**Gender**	**Titer by HI**	**Titer by MN**	**Person type**	**City**
1	31	2013.7.12	Male	1:20	0	Zoo worker	Guangzhou
2	27	2013.7.12	Male	1:40	1:40	Veterinary	Guangzhou
3	35	2013.8.4	Male	1:20	0	Slaughterhouse worker	Guangzhou
4	46	2013.8.4	Male	1:40	1:80	Poultry worker	Guangzhou
5	20	2013.8.4	Male	1:20	0	Poultry worker	Guangzhou
6	32	2013.7.16	Male	1:20	0	Retailer^a^	Guangzhou
7	26	2013.7.16	Male	1:20	1:20	Retailer	Guangzhou
8	25	2013.7.16	Female	1:40	1:40	Retailer	Guangzhou
9	35	2013.9.2	Male	1:20	0	Veterinary	Guangzhou
10	62	2013.5.15	Male	1:20	0	Swine farm worker	Guangzhou
11	38	2013.7.18	Male	1:20	0	Poultry worker	FoShan
12	26	2013.7.18	Male	1:20	1:20	Poultry worker	FoShan
13	45	2013.6.2	Male	1:20	0	Retailer	FoShan
14	47	2013.6.2	Female	1:20	1:20	Retailer	FoShan
15	30	2013.9.15	Male	1:20	0	Poultry worker	Jiangmen
16	52	2013.9.15	Male	1:20	0	Retailer	Jiangmen
17	47	2013.9.15	Male	1:20	0	Poultry worker	Jiangmen
18	41	2013.7.14	Male	1:20	0	Poultry worker	Qingyuan
19	17	2013.7.15	Male	1:20	1:20	Retailer	Qingyuan
20	50	2013.6.12	Male	1:20	0	Poultry worker	Zhongshan
21	27	2013.6.12	Male	1:20	0	Veterinary	Zhongshan

^a^Retailer in agricultural-trade market. HI, horse RBC hemagglutination inhibition assay; MN, microneutralization assay. H10N8 = A/Jiangxi-Donghu/346/2013(H10N8).

## Discussion

In recent years, a number of zoonotic influenza viruses causing human disease have been documented in China. In 1997, high-pathogenic avian influenza H5N1 infection occurred in Hong Kong (near Guangdong Province) and more than 70 human cases with 50% mortality have since then been reported in mainland China [15]. Since 2013, at least 365 people have been infected with avian H7N9 influenza virus in China, leading to 111 deaths [7]. Unlike the numerous research studies regarding highly pathogenic avian H5 and H7 influenza viruses, there have been relatively few detections of H10 influenza infections in both avian and other species. Similar to the H7N9 influenza virus, poultry are not thought to be clinically affected by the H10N8 virus. We are not aware of other reports showing increased risk of H10N8 infection among poultry workers; we have seen such reports regarding H7N9 infection. One study in Zhejiang province showed that 1.3% of poultry workers were H7N9 positive by HI in April and May 2013 [16]. Another study identified a substantial increase in seroprevalence of antibody against H7N9 among poultry workers in Shenzhen city, Guangdong Province, from 7.2% to 14.9% between May and December 2013 [17].

Our retrospective serologic study is the first to document serological evidence of human asymptomatic infection with H10N8 influenza virus among animal workers before 30 November 2013 (the time of the first index human H10N8 AIV case [18]) and may prove to be important in considering the origins of the H10N8 virus. More importantly, these seroepidemiological findings are corroborated by our previous epidemiologic study which suggested that H10N8 was recently circulating among poultry in Guangdong Province [3]. On the other hand, the evidence of infection involved only three of the 827 study subjects and all had relatively low HI and MN antibody titers (1:40 or 1:80). However, the subjects with elevated titers had clear exposures to animals and such low titers are consistent with other subclinical AIV infections in humans which may generate relatively lower antibody response [19]. Furthermore, these cross-sectional results could reflect the gradual decline of antibody titers against novel H10N8 as the date of infection is not known [20].

The results of the present study suggest that workers occupationally exposed to poultry may be at risk of H10N8 AIV infection. It is reassuring that more animal workers were not found to be seropositive and that these findings are consistent with other studies of H7N9 and H5N1 infections among China’s poultry workers. However, it seems appropriate that more extensive serological investigations regarding asymptomatic or subclinical H10N8 infections should be performed among groups expected to be at high risk (such as animal workers and healthcare workers).

## Conclusions

Study data suggest that animal workers may have been infected with the H10N8 virus before November 30, 2013, when the first human H10N8 case was recognized. It seems prudent to continue surveillance for H10N8 viruses among animal workers.

### Ethical approval

This study protocol was reviewed and approved by the Institutional Review Board at the Guangdong Center for Disease Control and Prevention.

## Abbreviations

AIV: avian influenza virus
HI: hemagglutination inhibition
MN: microneutralization

## References

[1] Shortridge KF, Peiris JS, Guan Y (2003). The next influenza pandemic: lessons from Hong Kong. J Appl Microbiol.

[2] Su S, Zhou P, Fu X, Wang L, Hong M, Lu G, Sun L, Qi W, Ning Z, Jia K, Yuan Z, Wang H, Ke C, Wu J, Zhang G, Gray GC, Li S (2014). Virological and epidemiological evidence of avian influenza virus infections among feral dogs in live poultry markets, China: a threat to human health?. Clin Infect Dis.

[3] Jiao P, Cao L, Yuan R, Wei L, Song Y, Shen D, Gong L, Luo K, Ren T, Liao M (2012). Complete genome sequence of an H10N8 avian influenza virus isolated from a live bird market in Southern China. J Virol.

[4] Su S, Qi W, Chen J, Cao N, Zhu W, Yuan L, Wang H, Zhang G (2012). Complete genome sequence of an avian-like H4N8 swine influenza virus discovered in southern China. J Virol.

[5] Jiao P, Yuan R, Wei L, Jia B, Cao L, Song Y, Gong L, Xu C, Ren T, Liao M (2012). Complete genomic sequence of a novel natural recombinant H6N2 influenza virus from chickens in Guangdong, Southern China. J Virol.

[6] Gao R, Cao B, Hu Y, Feng Z, Wang D, Hu W, Chen J, Jie Z, Qiu H, Xu K, Xu X, Lu H, Zhu W, Gao Z, Xiang N, Shen Y, He Z, Gu Y, Zhang Z, Yang Y, Zhao X, Zhou L, Li X, Zou S, Zhang Y, Li X, Yang L, Guo J, Dong J, Li Q (2013). Human infection with a novel avian-origin influenza A (H7N9) virus. N Engl J Med.

[7] WHO: **Number of confirmed human cases of avian influenza A(H7N9) reported to WHO.** [http://www.who.int/influenza/human_animal_interface/influenza_h7n9/Data_Reports/en/]

[8] Report of Health and Family Planning Commission of Jiangxi Province: **The third human case of H10N8 bird flu was confirmed.** [http://www.jxwst.gov.cn/gzdt/201402/t20140213_308109.htm] In Chinese.

[9] Qi W, Zhou X, Shi W, Huang L, Xiao X, Xia W, Liu D, Li H, Chen S, Lei L, Cao L, Wu J, He F, Song W, Li Q, Li H, Liao M, Liu M (2014). Genesis of the novel human-infecting H10N8 influenza virus and potential genetic diversity of H10N8 in poultry, China. Euro Surveill.

[10] García-Sastre A, Schmolke M (2014). Avian influenza A H10N8—a virus on the verge?. Lancet.

[11] To K, Tsang A, Chan J, Cheng V, Chen H, Yuen K (2014). Emergence in China of human disease due to avian influenza A (H10N8)—cause for concern. J Infect.

[12] Su S, Qi W, Zhou P, Xiao C, Yan Z, Cui J, Jia K, Zhang G, Gray G, Liao M, Li S (2014). First evidence of H10N8 avian influenza virus infections among feral dogs in live poultry markets in Guangdong Province, China. Clin Infect Dis.

[13] WHO: **Serological detection of avian influenza A(H7N9) virus infections by modified horse red blood cells haemagglutination-inhibition assay.** 2013. [http://www.who.int/influenza/gisrs_laboratory/cnic_serological_diagnosis_hai_a_h7n9_20131220.pdf]

[14] WHO Global Influenza Surveillance Network: **Manual for the laboratory diagnosis and virological surveillance of influenza.** 2011. [http://www.who.int/influenza/gisrs_laboratory/manual_diagnosis_surveillance_influenza/en/]

[15] **From the Centers for Disease Control and Prevention. Update: isolation of avian influenza A(H5N1) viruses from humans--Hong Kong, 1997–1998.** *JAMA* 1998, **279:**347–348. 9459456

[16] Yang S, Chen Y, Cui D, Yao H, Lou J, Huo Z, Xie G, Yu F, Zheng S, Yang Y, Zhu Y, Lu X, Liu X, Lau S, Chan J, To K, Yuen K, Chen H, Li L (2014). Avian-origin influenza A(H7N9) infection in influenza A(H7N9)-affected areas of China: a serological study. J Infect Dis.

[17] Wang X, Fang S, Lu X, Xu C, Cowling BJ, Tang X, Peng B, Wu W, He J, Tang Y, Xie X, Mei S, Kong D, Zhang R, Ma H, Cheng J (2014). Seroprevalence to avian influenza A(H7N9) virus among poultry workers and the general population in Southern China: a longitudinal study. Clin Infect Dis.

[18] Chen H, Yuan H, Gao R, Zhang J, Wang D, Xiong Y, Fan G, Yang F, Li X, Zhou J, Zou S, Yang L, Chen T, Dong L, Bo H, Zhao X, Zhang Y, Lan Y, Bai T, Dong J, Li Q, Wang S, Zhang Y, Li H, Gong T, Shi Y, Ni X, Li J, Zhou J, Fan J (2014). Clinical and epidemiological characteristics of a fatal case of avian influenza A H10N8 virus infection: a descriptive study. Lancet.

[19] Fox J, Cooney M, Hall C, Foy H (1982). Influenza virus infections in Seattle families, 1975–1979. II. Pattern of infection in invaded households and relation of age and prior antibody to occurrence of infection and related illness. Am J Epidemiol.

[20] Gerhard W (2001). The role of the antibody response in influenza virus infection. Curr Top Microbiol Immunol.

